# Surviving the Patchwork: Habitat Preferences of a Threatened Amphibian in a Fragmented Tropical Landscape

**DOI:** 10.1002/ece3.72785

**Published:** 2026-01-01

**Authors:** N. V. Rajiv, Vishnupriya Sankararaman, Vivek Ramachandran

**Affiliations:** ^1^ Wildlife Biology and Conservation Program National Centre for Biological Sciences, NCBS TIFR Bengaluru India; ^2^ Wildlife Conservation Society India Bengaluru India

**Keywords:** Agroforest, EDGE species, freshwater, GLMM, habitat preference, Kottigehara dancing frog

## Abstract

Tropical freshwater ecosystems are severely threatened, endangering evolutionarily distinct and globally endangered species the most. Understanding the habitat preferences of these vulnerable species is crucial for effective conservation. This study investigated the habitat associations of *Micrixalus kottigeharensis* , an evolutionarily distinct and globally endangered frog, within a mixed‐use landscape in the Western Ghats biodiversity hotspot. We surveyed 67 stream segments across 2500 ha, recording species count and habitat variables, and analysed these relationships using a Generalised linear mixed modelling framework. Our findings showed that stream hydrology, microclimate and land‐use together influenced the habitat preferred by *M. kottigeharensis* . They preferred streams with a greater percentage of emergent substrate and dense canopy cover, emphasising the importance of structurally complex habitats with healthy riparian vegetation. Stream morphology was also an important characteristic, as they preferred riffles and braided channels over other stream types. Furthermore, in the landscape context, they were more abundant in grasslands and forests compared to intensively managed plantations. We also note that their peak activity was observed during the post‐monsoon season. These results highlight the critical role of specific habitats in determining the distribution of *M. kottigeharensis* . Additionally, they provide valuable insights, especially for landowners and managers of agroforests, for targeted habitat management and restoration strategies such as maintaining the natural stream flow, prevention of stream channelisation and mining, and nurturing riparian buffers, aimed at conserving this endangered species.

## Graphical abstract

This study investigated the habitat preferences of *Micrixalus kottigeharensis* , an evolutionarily distinct and globally endangered frog, within a mixed‐use landscape in the Western Ghats biodiversity hotspot. Our results showed that stream hydrology, microclimate and land‐use together influenced the habitat preferred by *M. kottigeharensis* . These results provide crucial evidence for habitat management and restoration strategies aimed at protecting threatened freshwater amphibians in dynamic stream ecosystems.

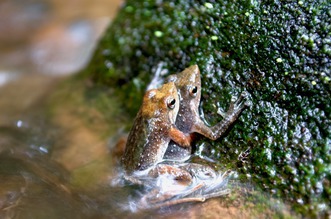

## 1 Introduction

Freshwater ecosystems harbour a significant share of the global biodiversity despite occupying < 1% of the earth's surface (Dudgeon et al. 2006; Balian et al. 2008). Yet, freshwater habitats and species are among the most threatened, having experienced an 85% decline in populations between 1970 and 2020 (WWF International 2024). Studies indicate that among freshwater vertebrates, amphibians are the most threatened, facing unprecedented rates of global declines and extinctions, particularly in the tropics (Dudgeon et al. 2006; Luedtke et al. 2023).

The primary drivers of amphibian decline include disease, climate change effects, habitat loss or degradation and over‐exploitation. Among the multiple convergent stressors that are driving their decline, habitat degradation is the most severe (Blaustein et al. 2011; Luedtke et al. 2023). Agriculture, timber and plant harvesting, infrastructure development, pollution, mining, water management and human disturbance are the underlying causes of amphibian habitat degradation. The Western Ghats (India), a UNESCO World Heritage Site (UNESCO 2012), is one such region with a significantly high number of threatened freshwater species (Sayer et al. 2025). It is recognised as a global biodiversity hotspot of high endemism (Myers et al. 2000) and has a remarkably high species richness of globally threatened amphibians (Luedtke et al. 2023).

The anuran family of Micrixalidae is a Western Ghats region endemic containing a single genus, *Micrixalus*, an ancient frog lineage, which evolved more than 60 million years ago (Roelants et al. 2004). Since it has very few close relatives, the loss of these species will result in a disproportionate loss of evolutionary diversity. Literature states that such evolutionarily distinct amphibians decline disproportionately when their habitats are subject to long‐term modifications (Greenberg et al. 2018). Hence, understanding their habitat and its threats is one of the necessary measures for the persistence of these amphibians.

**FIGURE 1:**
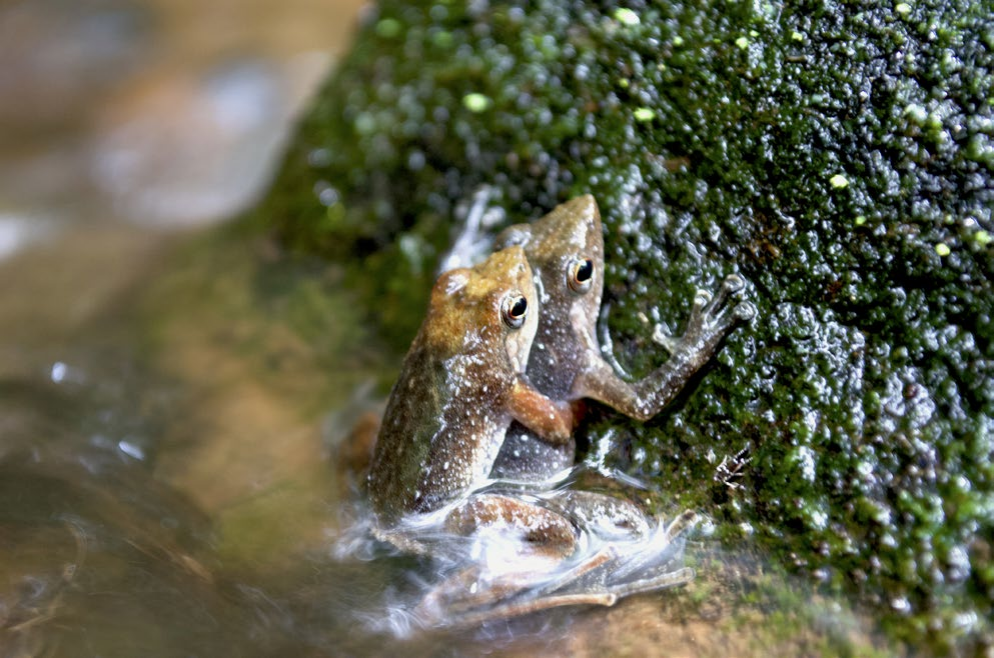
A pair of *M. kottigeharensis* (Kottigehara dancing frog) in amplexus in a central Western Ghats stream. Image by N. V. Rajiv.

**FIGURE 2:**
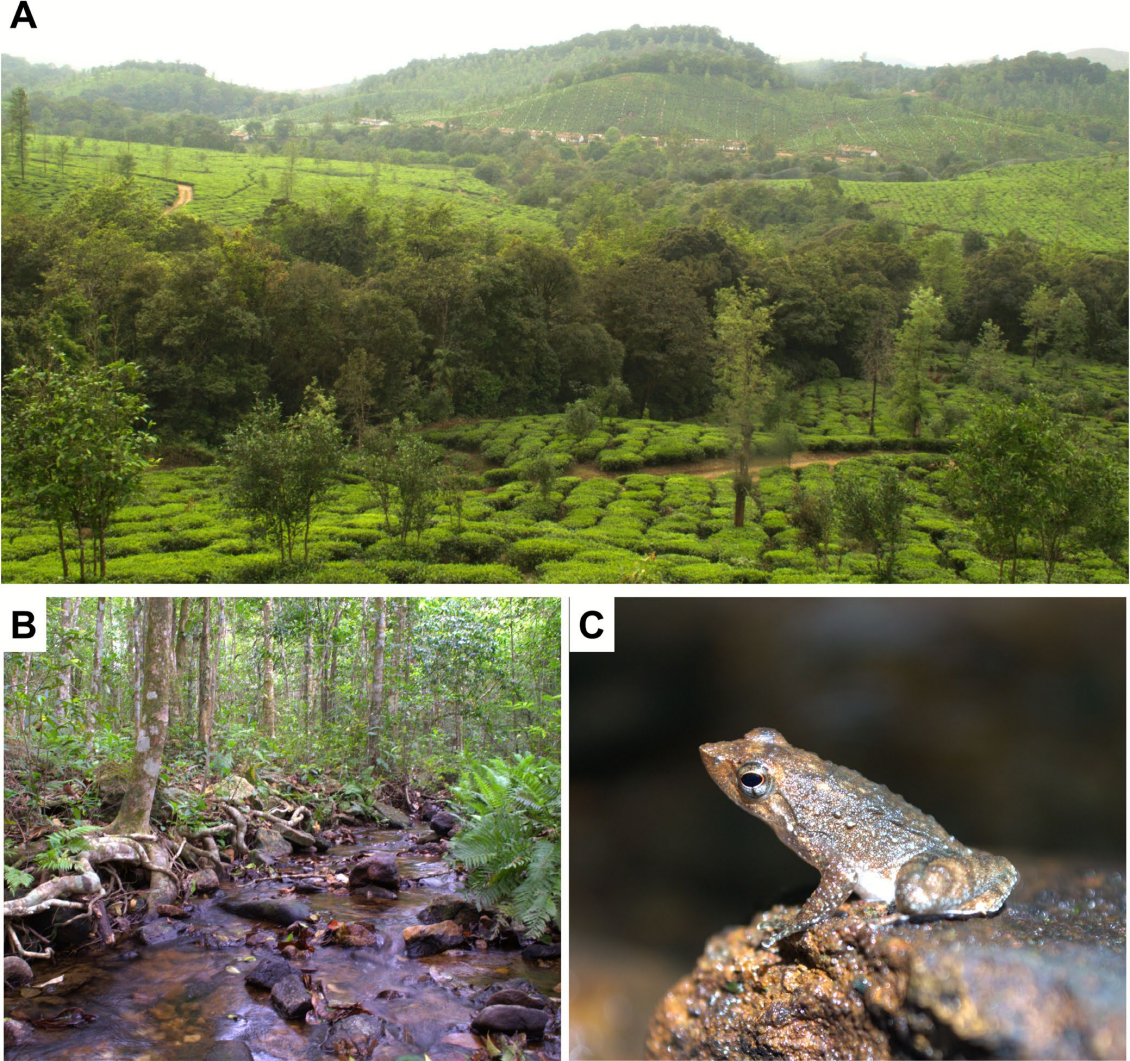
(A) Study area with mixed‐use landscape consisting of plantations and forest patches. The forest line between the plantation are streams with riparian vegetation. (B) Stable stream habitat with healthy riparian vegetation where *M. kottigeharensis* occurs. (C) *Micrixalus kottigeharensis* (Kottigehara dancing frog). Images by N. V. Rajiv.

This study focuses on one such threatened endemic species, *Micrixalus kottigeharensis* (Rao 1937) (Kottigehara Dancing Frog) (Figure 1 and Figure 2C), for which detailed ecological studies are scarce. It is classified as Vulnerable according to IUCN's Red List criteria (IUCN SSC Amphibian Specialist Group 2021) and is distributed only in the central Western Ghats (Biju et al. 2014). *M. kottigeharensis* prefers fast‐flowing streams of evergreen forests of Western Ghats (Figure 2B) and is also found in Myristica swamps of Karnataka (Biju et al. 2014; Mudke et al. 2020).

The threat to freshwater streams occupied by *M. kottigeharensis* is higher in human‐use landscapes, given the drastic land‐use change unfolding in the Western Ghats (Jha et al. 2000). The ongoing changes to global climate are likely to further intensify these threats to freshwater ecosystems (Markovic et al. 2017). Yet there is very little information available on the habitat preference of the threatened endemic *M. kottigeharensis* , while the existing studies focus on its communication and behaviour (Preininger, Boeckle, et al. 2013; Preininger, Stiegler, et al. 2013), physiology (Biju et al. 2014) and natural history (Mudke et al. 2020). Investigation of habitat preferences can provide crucial inputs to habitat‐based conservation efforts that are tailored to the requirements of a specific species (Barton et al. 2024). Thus, this study aimed to understand the habitat characteristics of *M. kottigeharensis* within mixed‐use landscapes.

Males of *Micrixalus Kottigeharensis* perform foot‐flagging while perched on stones (Gururaja 2010; Biju et al. 2014), whereas females deposit eggs in shallow, flowing sections of perennial streams (Mudke et al. 2020). Therefore, stream hydrology appears to influence its selection of breeding sites. Being a diurnal species, they are particularly susceptible to desiccation, and canopy cover is likely to provide the microclimatic conditions necessary for its survival. Habitat degradation from stream fragmentation, pollution and riparian vegetation loss in mixed‐use landscapes constitutes a major threat to this species. Consequently, land‐use is expected to be a key determinant of its distribution. Based on these ecological observations, we expected that stream hydrology, microclimatic conditions and surrounding land‐use would play a significant role in shaping the habitat preference of this species. Accordingly, our research sought to determine the individual effects of stream hydrology, microclimate and land‐use on *M. kottigeharensis* count and to evaluate whether combined predictors explain variation in counts more effectively than individual factors (Table 1).

**TABLE 1:** Multiple a priori hypotheses to determine the habitat preferences of *M. kottigeharensis* .

Hypothesis group	Hypothesis	Covariates
Hydrology (H1)	Frog count increases with favourable stream hydrological conditions.	% emergent‐substrate, Stream‐section‐type (riffle, run, pool, braided, step‐pool), slope
Microclimate (H2)	Microclimate determines frog count.	Canopy cover
Land‐use (H3)	Land‐use influences frog counts with intensively managed land‐use having a negative impact on them.	Land‐use
Hydrology + Microclimate	Combined effects of hydrology and microclimate explain count better than either alone.	All H1 + H2 covariates
Hydrology + Land‐use	Hydrology and land‐use jointly influence frog count.	All H1 + H3 covariates
Microclimate + Land‐use	Microclimate and land‐use jointly influence frog count.	All H2 + H3 covariates
Hydrology + Microclimate + Land‐use	The combination of stream hydrology, microclimate and land‐use determine frog count.	All H1 + H2 + H3 covariates

*Note:* The covariates column lists the environmental variables measured to test the respective hypotheses.

## 2 Methods

### 2.1 Study Area

**FIGURE 3:**
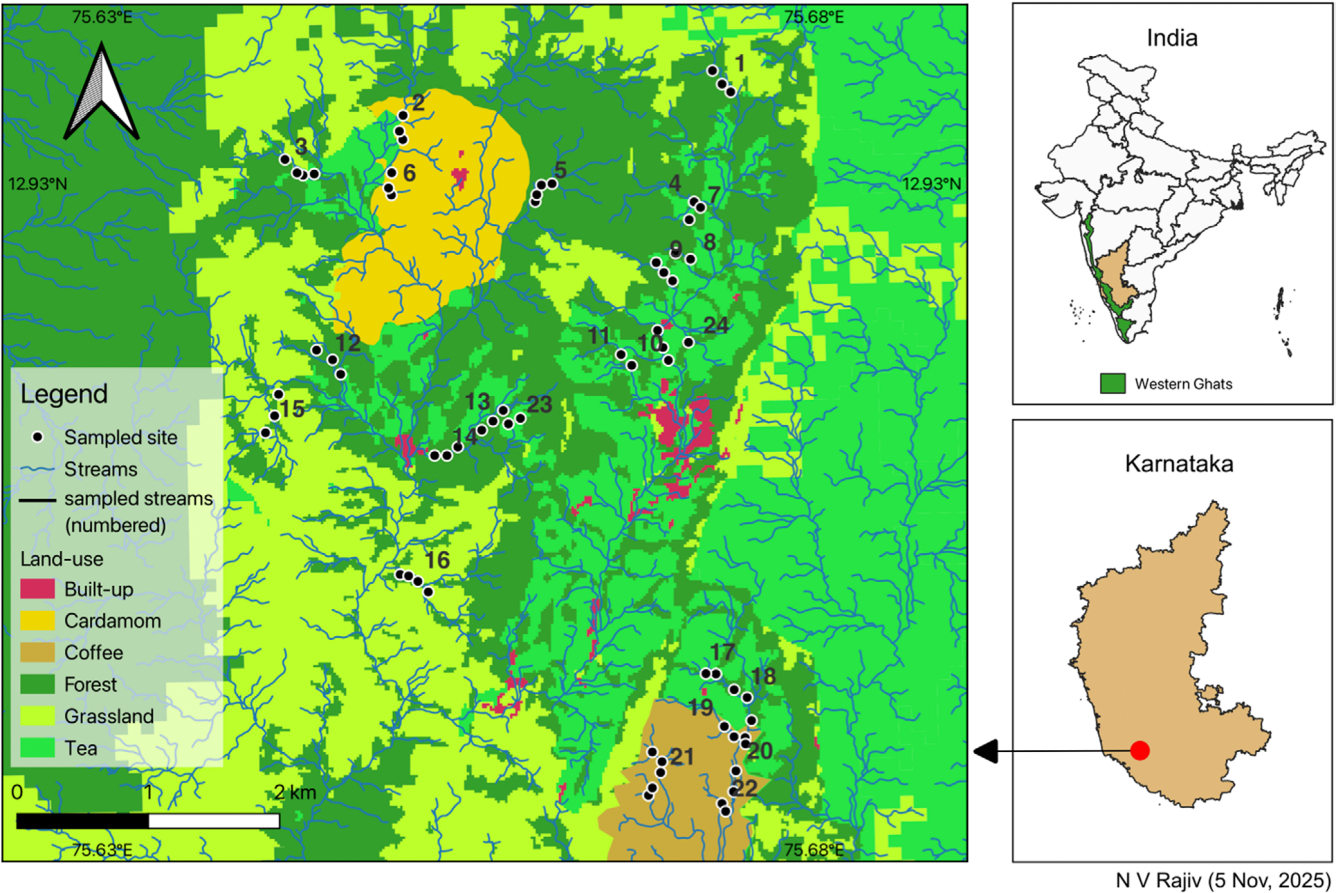
Left—Land‐use map of the study area along with the network of streams and sampled locations. Right—Map of India with Karnataka state, Western Ghats and study area marked.

We conducted this study in Kadamane, a mixed‐use, forest‐grassland‐plantation mosaic, spread over 2500 ha, in the central Western Ghats, India (12.919602, 75.668633). It is approximately 20 km away from the nearest town in Sakleshpur taluk and lies in Hassan district, Karnataka, India. It lies in proximity to two protected areas (PAs), the Kudremukh National Park (40 km north‐west) and Pushpagiri Wildlife Sanctuary (20 km south). This region receives an annual rainfall of over 6000 mm (Venkatesh et al. 2021), most of which is during the south‐west monsoon season (June–October) in India. The study area encompasses a variety of land‐use types, including grasslands, forest, tea plantations, abandoned coffee and cardamom plots, interspersed with human settlements (Figure 2A). With an elevation ranging from 585 to 1157 m above sea level (ASL), the area is drained by numerous perennial and seasonal streams which are a part of the Kempu hole river basin (Figure 3). The streams of the study area are key to freshwater security of the region. The decision to investigate the habitat preference of *M. kottigeharensis* in a mixed‐use agroforest landscape was based on a gap analysis (Scott and Schipper 2006), which showed that 75% of the confirmed occurrences of this species were outside the formal protected area network in the Western Ghats (GBIF.org 2024).

### 2.2 Field Methods

We used ASTER Global Digital Elevation Model (GDEM) Version 3 (ASTER Science Team 2019) in QGIS (v 3.34) (QGIS Development Team 2024) to delineate all streams of the study area using Strahler stream order (Strahler 1957). One third of the streams were randomly chosen and verified by direct observation. Finally, a total of 24 streams were selected after omission of inaccessible regions. We distributed 67 transects (sites) across 24 streams (Figure 3). Each transect was 10 m long and the width extended up to 5 m beyond one of the randomly chosen banks. Successive transects were separated by at least 80 m distance to ensure independence between sites.

We surveyed all 67 sites during each of the three distinct seasons (pre/onset‐monsoon, monsoon and post‐monsoon) between May and December 2024. Sampling comprised three seasons with two planned visits per season; however, due to logistical constraints, one season (pre/onset‐monsoon) was sampled only once at each site. A gap of at least 1 month was ensured between successive surveys at the same site. Since *M. kottigeharensis* is a diurnal species (Biju et al. 2014), we sampled between 8:00 and 18:00 h. At each site, two surveyors searched the stream and the adjacent riparian habitat for a fixed duration of 15 min, and the observed number of *M. kottigeharensis* individuals was recorded. *M. kottigeharensis* was identified based on its call (Krishna and Krishna 2006) and physical characteristics (Biju et al. 2014). We measured ecologically relevant habitat variables (covariates column in Table 1) to capture the hydrology, microclimate and land‐use of the surveyed sites. GPS coordinates and elevation of each site were recorded using Garmin GPS etrex10 (Garmin Ltd.). Canopy cover was measured using a densiometer. The slope of a site was calculated using QGIS's core terrain analysis tool (using GDEM with 30 m resolution). The *percentage of* emergent substrate in the stream section of a site was estimated using the line intercept method (Bonham 2013). Distance to the road was measured using QGIS tools and used as a potential covariate to control sampling bias (towards accessible streams). Land‐use was calculated for a circle with a 100 m diameter centred on a stream site (Strayer et al. 2003; Damanik‐Ambarita et al. 2018). We used the 2005 LULC classification (Roy et al. 2016) and supplemented it with ground surveys to determine the land‐use. When multiple land‐use types were present within the circle, the dominant land‐use (occupying the largest proportion of area in the circle) was chosen.

### 2.3 Analysis

We analysed the maximum count of *M. kottigeharensis* per site as the response variable and tested its association with habitat covariates as predictors. Maximum count is the highest frog count per site within a season (two survey visits in a season). Maximum count was chosen over average count as it is more robust to missed detections on some visits (Rigby and Johnson 2025). The sites' distance to the closest road was initially included as a potential accessibility covariate. However, exploratory analysis revealed that it was not related to *M. kottigeharensis* count and was therefore excluded from the covariates list.

We used an information‐theoretic approach in modelling (Burnham and Anderson 2002) and built multiple a priori models based on the natural history of the species and past fieldwork experience. We defined eight a priori candidate models representing hydrology, microclimate and land‐use hypothesis (Table 2). We built Generalised linear mixed models (GLMMs) using fixed effect habitat covariates that represented our a priori hypothesis. Since the data were collected over three different seasons of the year, we accounted for the potential similarity in season‐wise data using a temporal grouping random effect covariate (season) in GLMM (Bolker et al. 2009; Zuur et al. 2009). No spatial autocorrelation was observed on both frog count data and model residuals (Moran's *I* ≈ 0, *p* > 0.05 across all seasons).

**TABLE 2:** Model selection results for generalised linear mixed modelling analysis of habitat preference of *M. kottigeharensis* .

	Model	log(*L*)	*K*	AIC_*c*_	∆_*i*_	*w*_*i*_
**1**	**Global hydrology + microclimate + land‐use**	**−381.80**	**14**	**793.85**	**0.00**	**0.99**
2	Hydrology + microclimate	−391.40	10	803.96	10.11	0.01
3	Hydrology + land‐use	−388.80	13	805.55	11.70	0.00
4	Microclimate + land‐use	−401.07	8	818.90	25.05	0.00
5	Hydrology	−401.35	9	821.64	27.79	0.00
6	Microclimate	−407.93	4	824.06	30.21	0.00
7	Land‐use	−414.37	7	843.32	49.47	0.00
8	Null	−423.21	3	852.53	58.68	0.00

*Note:* The table lists candidate models, ranked in increasing order of ∆_*i*_. The null model contains no fixed effect predictors. All the above models have season as the random effect predictor. The best‐fit model is bolded. Model components are as follows: value of the maximised log‐likelihood function (log(*L*)), number of estimated parameters (*K*), small‐sample corrected Akaike Information Criterion (AIC_*c*_), Difference between current model AIC_*c*_ and lowest AIC_*c*_ model values (∆_*i*_), Akaike weight (*w*_*i*_).

All covariates were standardised by subtracting the mean and dividing by the standard deviation (Rhodes et al. 2009) as part of statistical preprocessing. Collinearity was tested among all covariates using the variance inflation factor (VIF > 5 considered high collinearity) and a correlation matrix. To begin with, we fit a Poisson distribution for the count data. However, the Poisson model showed clear signs of over‐dispersion; hence, we chose negative binomial as the error distribution.

We used the glmmTMB package (Brooks et al. 2024) in the R statistical program to run GLMMs. Model fit was assessed using simulation‐based residual checks and variance‐based measures.

### 2.4 Generalised Linear Mixed Model (GLMM)

We use *Y*_*ik*_ as the maximum observed count for site *i*, during season *k*, with the error distribution *Y*_*ik*_ ∼ Negative Binomial(*μ*_*ik*_,*θ*). *μ*_*ik*_ is the expected count of *M. kottigeharensis* , and *θ* is the dispersion parameter for a negative binomial distribution. The global model with fixed effect covariates and random effects having a negative binomial error distribution and logarithmic link function is as shown below:

(1) $$\begin{array}{cc} \log\left(\mu_{\mathrm{ijk}}\right) & =\beta_{0}+{\beta_{1}}^{*}\;\text{canopy}+{\beta_{2}}^{*}\;\text{emergent}\_\text{substrate}\\ & +{\beta_{4}}^{*}\;\text{stream}\_\text{braided}+{\beta_{5}}^{*}\;\text{stream}\_\text{pool}\\ & +{\beta_{6}}^{*}\;\text{stream}\_\text{riffle}+{\beta_{7}}^{*}\;\text{stream}\_\text{step}\_\text{pool}\\ & +{\beta_{8}}^{*}\;\text{slope}+{\beta_{9}}^{*}\;\text{landuse}\_\text{coffee}+{\beta_{10}}^{*}\;\text{landuse}\_\mathrm{tea}\\ & +{\beta_{11}}^{*}\;\text{landuse}\_\text{forest}+{\beta_{12}}^{*}\;\text{landuse}\_\text{grassland}\\ & +{\beta_{13}}^{*}\;\text{landuse}\_\text{cardamom}+v_{k} \end{array}$$

where *β*_0_ is the intercept, and *β*_1–13_ are the fixed effect coefficients for the predictors. The stream type—run is chosen as the reference level. Abandoned cardamom is chosen as the reference for land‐use. *v*_*k*_ ∼ *N*(0,*σ* ^2^_season_) captures the random variation among seasons.

### 2.5 Model Selection

No multicollinearity was detected between predictor variables (VIF < 3). There was no evidence of additional overdispersion beyond that modelled by *θ* parameter in negative binomial GLMM (Pearson chi‐squared/residual degrees of freedom; *ĉ* = 0.9) The ratio of sample size to estimated parameters (*n/K*) was < 40 for the global model; hence, model selection was based on small‐sample corrected Akaike Information Criterion (AIC_*c*_) (Burnham and Anderson 2002). Candidate models were ranked by AIC_*c*_, and the difference between each model and the lowest AIC_*c*_ value (Δ_*i*_) was calculated. Models with Δ_*i*_ ≤ 2 were considered strongly supported, while those with Δ_*i*_ > 10 indicated negligible support. The Akaike weight (*w*_*i*_) was also calculated to assess the relative support for each model within the candidate set. Parameter estimates and their 95% confidence intervals from the top model(s) (Δ_*i*_ ≤ 2) were used to infer the relative importance and the direction of covariates on count.

## 3 Results

**FIGURE 4:**
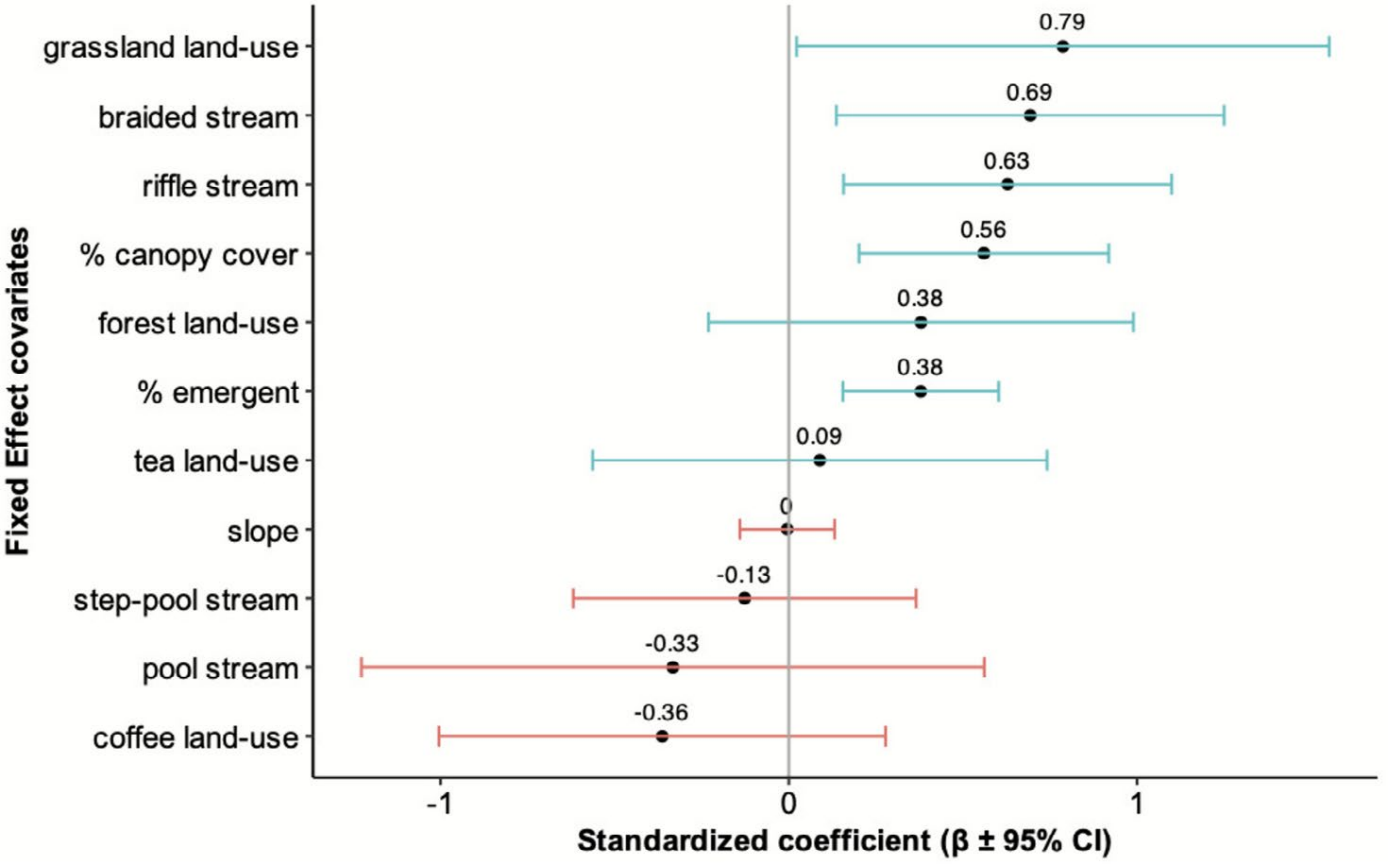
Results from the global negative binomial mixed model (log link) showing standardised regression coefficients (*β* ± 95% CI), which depict the relative influence of hydrology, microhabitat, and land‐use covariates on frog counts. Estimates correspond to those in Table 3. The horizontal bars of each predictor encompass the 95% Confidence Interval (CI). A blue bar signifies a positive association, and a red bar signifies a negative association between the response variable (frog count) and the predictor.

Variation in *M. kottigeharensis* count was best explained by hydrological, microclimatic and land‐use conditions acting together. Model selection (based on AIC_*c*_) identified the global model, containing all three covariate groups, as the best supported model (∆AIC_*c*_ = 0, AIC_*c*_ weight = 0.99) (Table 2). All competing models had ∆AIC_*c*_ > 10, indicating negligible support; therefore, subsequent inference was based on the global model. The estimated coefficients and their 95% confidence intervals for the final model are shown in Table 3 and illustrated in Figure 4. Simulated residuals did not deviate from a uniform distribution (DHARMa Kolmogrov‐Smirnov test, *p* = 0.25) and there was no evidence of overdispersion (*ϕ* = 0.9), indicating that the global negative binomial model was an adequate fit. The marginal *R* ^2^ (variance explained by fixed effects alone) was 0.43, while the conditional *R* ^2^ (variance explained by both fixed effects and random effects) was 0.76.

**TABLE 3:** Results of generalised linear mixed model explaining the variation in the count of *M. kottigeharensis*.

**Fixed effects**	**Estimate**	**Standard error**
(Intercept)	0.137	0.625
% canopy cover	0.560	0.183
% emergent	0.379	0.114
Braided stream	0.693	0.284
Pool stream	−0.333	0.456
Riffle stream	0.628	0.240
Step‐pool stream	−0.127	0.251
Coffee land‐use	−0.363	0.327
Forest land‐use	0.380	0.311
Grassland land‐use	0.787	0.390
Tea land‐use	0.089	0.333
Slope	−0.004	0.069
**Random effects**	**Variance**	**SD**
Season (Intercept)	0.78	0.88

*Note:* These results pertain to the final model (Bolded in Table 2). The table details the fixed and random effect influence on the response variable (count of *M. kottigeharensis* per site). No. of obs: 201, groups: season = 3.

### 3.1 Fixed Effects

**FIGURE 5:**
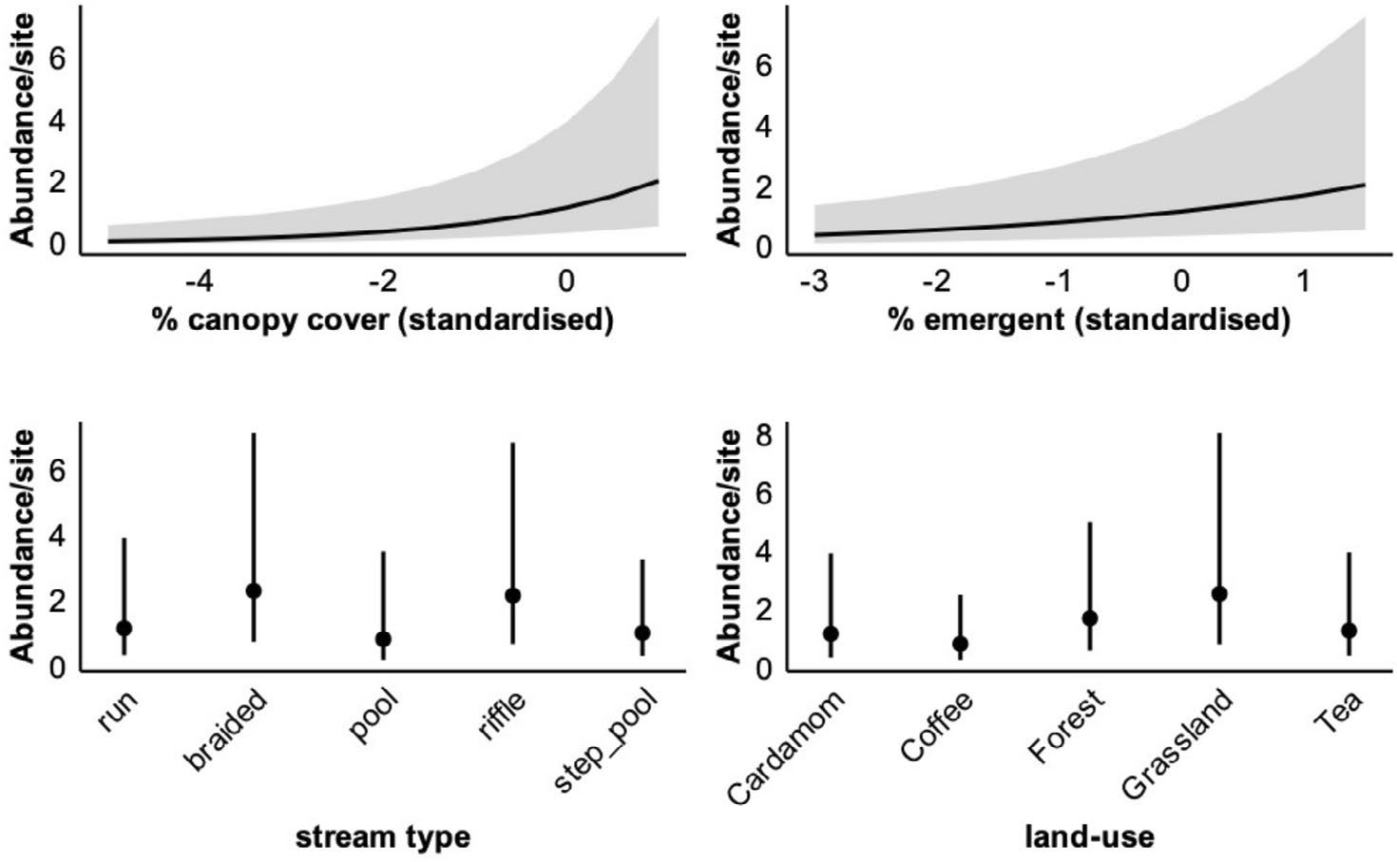
Predicted abundance of *M. kottigeharensis* across (a) standardised percentage canopy cover, (b) standardised percentage emergent substrate, (c) stream section type, and (d) land‐use. The 95% confidence interval is represented by shaded regions (top graphs) and vertical lines (bottom graphs). Predictions were generated using fixed effect estimates while holding other covariates at their mean values.

The marginal *R* ^2^ value indicated that the fixed effect ecological predictors explain a substantial share of the variation in count. Microclimate, represented by canopy cover, was positively associated with *M. kottigeharensis* count (*β* = 0.56 ± (SE) 0.18). Hydrological features, particularly the availability of emergent substrate within the stream, and the stream section type influenced their numbers. Emergent substrate availability was positively associated with count (*β* = 0.38 ± (SE) 0.11). Among stream section types, braided (*β* = 0.69 ± (SE) 0.28) and riffle (*β* = 0.63 ± (SE) 0.24) sections supported higher frog count relative to the run reference category. Variation in *M. kottigeharensis* count was further influenced by the surrounding land‐use type. Grassland sites showed higher counts relative to abandoned cardamom (reference category) (*β* = 0.79 ± (SE) 0.39), indicating a positive association with this land‐use type. Although forest sites showed moderate positive associations (*β* = 0.38 ± (SE) 0.31), the relatively large standard error suggests some uncertainty in this effect. Coffee plantations showed a negative association with frog count (*β* = −0.36 ± (SE) 0.38), while tea plantations had near‐zero association (*β* = −0.36 ± (SE) 0.38); however, in both cases, the wide standard errors indicated considerable uncertainty and weak support for these effects. Overall, these results suggest that natural habitats such as grasslands and forests support higher *M. kottigeharensis* count than intensively managed plantations. Model predictions further indicate that hydrological, microclimatic and land‐use factors collectively influenced the species' abundance patterns, as illustrated in the prediction plots (Figure 5).

### 3.2 Random Effects

**FIGURE 6:**
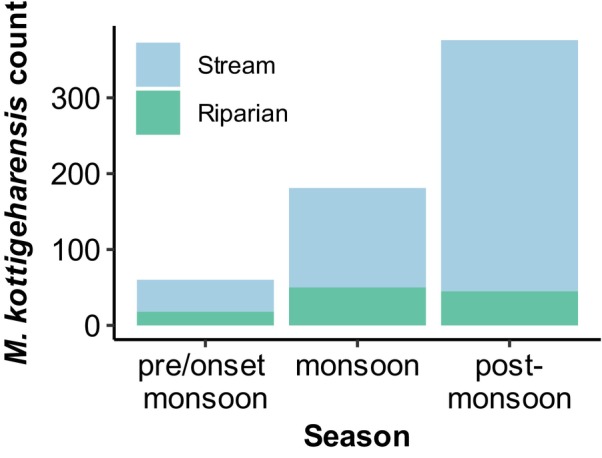
Temporal trends in *M. kottigeharensis* count. Stream and riparian represent the habitat where the species was detected. pre/onset‐monsoon = May–June; monsoon = July–Sep; post‐monsoon = Oct–Dec in the year 2024.

**FIGURE 7:**
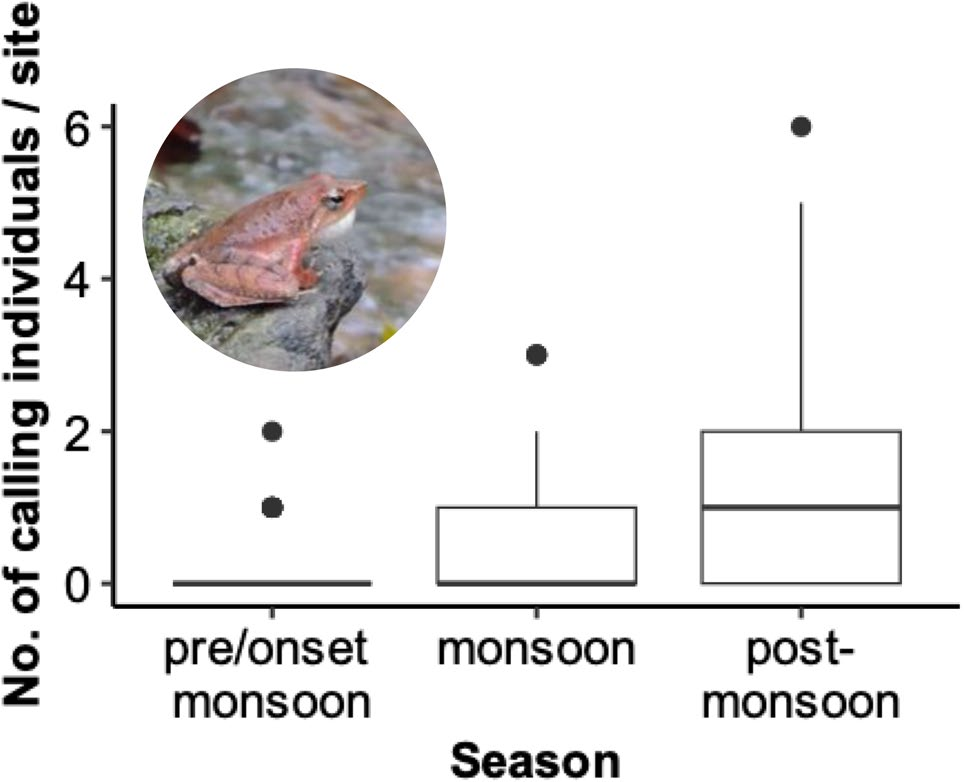
Box plot depicting the number of calling individuals of *M. kottigeharensis* , per site, across a temporal scale. pre/onset‐monsoon = May–June; monsoon = July–Sep; post‐monsoon = Oct–Dec in the year 2024.

The random effect variable (season), representing inter‐season variability in frog count, contributed moderately to the overall variance (variance ± SD = 0.78 ± 0.88). We analysed the seasonal variation in frog count and observed that they were most abundant in the post‐monsoon season (Oct–Dec) in central Western Ghats (Figure 6). This peak in abundance was primarily driven by an increase in the number of individuals recorded in the stream, whereas riparian abundance remained relatively stable across seasons (Figure 6). We analysed the variation in the number of calling individuals across seasons and present this result here (Figure 7). Nearly 71% of all calling individuals were detected in the post‐monsoon season (Oct–Dec), suggesting that this period aligns with the species' breeding activity.

## 4 Discussion

In the tropics, where freshwater species are severely threatened by habitat degradation and loss (WWF International 2024), reliable scientific knowledge of habitat preferences is crucial for developing effective conservation strategies for such species. This study employs a rigorous design and analytical framework to determine the habitat preference of a globally threatened and evolutionarily distinct amphibian. Our results showed that *M. kottigeharensis* abundance was best explained by a combination of hydrological, microclimatic and land‐use characteristics. We find that streams with a higher percentage of emergent substrate and canopy cover supported greater numbers of individuals, highlighting the importance of structurally complex and shaded habitats. Furthermore, the species was more frequently associated with riffles and braided stream sections than other stream types. Land‐use also influenced the local abundance patterns suggesting that in‐stream habitat features and broader landscape context synergistically shaped suitable conditions for this stream‐dwelling frog. We also note that *M. kottigeharensis* was most active in the post‐monsoon season in Central Western Ghats, India, with significantly higher counts compared to other sampling occasions. These results provide crucial evidence for habitat management and restoration strategies aimed at protecting threatened freshwater amphibians in dynamic stream ecosystems.

### 4.1 Habitat Characteristics

The availability of suitable microhabitats influences amphibian distribution and abundance at the local scale (Thorpe et al. 2018). The physical features of the streams, governed by hydrological processes, play an important role in creating habitats suitable for stream‐dwelling amphibians. Emergent substrate, in particular, which includes rocks and vegetation in streams, facilitates refuge, foraging and breeding, thereby providing important microhabitats for amphibians (Wells 2010; Burrow and Maerz 2022). Our findings showed that *M. kottigeharensis* count was higher at sites characterised by greater availability of emergent substrate in streams. We know that the frogs of the genus *Micrixalus* use emergent rocks as perching sites while foot‐flagging, which enhances conspecific signalling (Preininger, Stiegler, et al. 2013). While the primary utility of such substrates appears to aid signalling, their usage as hiding and shelter spots cannot be underestimated. Additionally, we observed a few individuals of this species feeding on flies and spiders on emergent rocks. Thus, emergent substrates in streams could play a multifunctional role in sustaining the populations of the focal frog species. Another important hydrological characteristic associated with *M. kottigeharensis* count was stream channel morphology. We find that their numbers were high in riffle and braided sections compared to other stream types. Elevated dissolved oxygen (DO) levels and diverse invertebrate prey in riffles contribute to higher abundance of amphibians in such sites (Gomez and Anthony 1996; Wells 2010). Braided streams are distinguished by the presence of multiple shallow channels that divide and rejoin around small islands (Leopold et al. 1964; Gerald and David 1996). Shallow water may be particularly suitable for the fossorial tadpoles of *M. kottigeharensis* (Senevirathne 2016; Mudke et al. 2020), while the islands formed in braided sections create additional refuges from predators and disturbances.

Riparian vegetation is a key component of a healthy freshwater ecosystem, supporting diverse amphibian species (Semlitsch and Bodie 2003). We used canopy cover as a critical indicator of riparian health since it influences microclimate, ensures stream bank stability and provides structurally complex habitats (Werner and Glennemeier 1999; Hughes 2016). Dense canopy cover was described as a significant characteristic of the habitat used by the frogs of the genus *Micrixalus* (Reddy et al. 2002; Biju et al. 2014). Our study confirmed these findings using a modelling approach, where an increase in canopy cover was associated with a higher number of *M. kottigeharensis* . The individuals of this species were detected not only within the stream channel but also in the surrounding riparian zones (Figure 6), highlighting the necessity of canopy‐dense habitats in supporting their presence. Dense canopy cover reduces the temperature fluctuations within streams (Skelly et al. 2002). These patterns highlight the multifaceted benefits of maintaining healthy riparian vegetation for stream‐dwelling amphibian populations. We recommend that future amphibian conservation plans should consider preserving existing riparian vegetation and target restoration along degraded riparian zones.

### 4.2 Spatial Influence

The habitat surrounding the sampling locations also influenced *M. kottigeharensis* count. Varying impacts of land‐use on the diversity and abundance of amphibian populations are well‐documented (Browne et al. 2009; Hansen et al. 2019). Our results suggest that forests and grasslands supported a higher number of individuals compared to intensively managed plantations. Although classified as grassland at the landscape scale, these areas form a mosaic of open grasslands interspersed with tropical evergreen forests in the valleys, where dense riparian vegetation persists. Therefore, the positive association between grassland and *M. kottigeharensis* abundance likely reflects the availability of forested riparian margins within these mosaics rather than a preference for open habitats. Given the relatively large confidence intervals for forest, coffee and tea land‐use categories, these effects should be interpreted with caution. Nevertheless, our findings reinforce that maintaining structurally intact riparian vegetation is critical for sustaining stream‐dwelling amphibian populations in the Western Ghats, where land‐use intensification in agroforests has been shown to disproportionately impact such taxa (Sankararaman and Miller 2024).

### 4.3 Temporal Factor

In all our models, season was used as a random effect variable to account for possible temporal grouping in the data (Zuur et al. 2009). This structure ensured that inference on fixed effects was not confounded by unmodelled temporal dependence. The observed variance in the random effect indicates that, after accounting for fixed effects related to microclimate, hydrology and land‐use, there remained moderate variability in counts attributed to seasonal differences. Such variations in random effects can inform key patterns of ecological heterogeneity (Harrison et al. 2018). Our results showed that *Micrixalus kottigeharensis* was most abundant and actively calling during the post‐monsoon season (Oct–Dec) (Figure 7), suggesting that this period corresponds to its peak breeding activity (Heard et al. 2015). A possible explanation for post‐monsoon breeding is the availability of stable hydrological conditions to maximise reproductive success of the species (Wells 2010; Winters et al. 2024). Elsewhere in Sundaland, reduced predation and interspecific competition were the reasons for post‐monsoon breeding in amphibians (Inger and Voris 2001). However, Reddy et al. recorded the maximum mean density of *M. saxicola* in the monsoon season (June–September) (Reddy et al. 2002). In conclusion, the occurrence of the target species was influenced by temporal factors with a clear preference for the post‐monsoon period.

### 4.4 Caveats

While this study provides valuable insights into how hydrology, microclimate and land‐use influence *M. kottigeharensis* abundance, certain methodological aspects warrant consideration. One logistical limitation of this study was that, despite planning two visits per season, the pre/onset monsoon season was sampled only once at each site. This resulted in unequal within‐season replication. Nevertheless, GLMMs accommodate such unbalanced designs, and the use of maximum count from repeated visits as the response variable further reduces the potential bias. Although the study did not explicitly account for detection probability, the use of maximum count across visits helped to minimise potential biases associated with imperfect detection. Moreover, the analysis focused on site‐level covariates that represent stable environmental features (hydrology, microclimate and land‐use), thereby reducing the influence of short‐term variation in detection. Consequently, the modelling framework provides meaningful insights into habitat‐abundance associations, even in the absence of explicit detection modelling.

### 4.5 Threats to Habitat

The microclimatic, hydrological and land‐use features that support *M. kottigeharensis* are under growing threat from human activities in mixed‐use areas. Channelisation, in‐stream mining, damming and water extraction are widespread in the hill streams of Western Ghats (Gadgil et al. 2011). These activities can significantly alter the physical structure of streams and alter their flow regimes, threatening the habitat of stream organisms (Allan 2004; Palmer et al. 2010). Moreover, the loss of riparian vegetation primarily due to agriculture and infrastructure development has undermined the ecological function of riparian zones, leading to degradation and loss of stream biota. Agricultural intensification in Western Ghats has been a major cause of habitat loss and fragmentation (Kale et al. 2016). These impacts are intensified when combined with climate change (Kale et al. 2016). Extreme weather events are likely to increase in severity and frequency, with a projected 10% increase in high‐intensity tropical cyclones (Intergovernmental Panel on Climate Change (IPCC) 2023). Changing temperature regimes, floods and droughts can further disrupt stream flow dynamics and accelerate habitat degradation. In summary, based on our findings, we note that the habitat preferred by *M. kottigeharensis* is under increasing threat from the combined effects of climate change and anthropogenic disturbances. Immediate conservation measures targeted at both the landscape‐scale and microhabitat‐level interventions are necessary to protect this endemic, threatened amphibian.

We propose a two‐fold strategy for the rejuvenation of stream habitats. Firstly, intact stream ecosystems must be protected from deforestation and other geomorphological changes such as damming and dredging. Secondly, degraded stream habitats must be restored using a natural design approach (Rosgen 2011). Protecting and managing freshwater streams is not only beneficial to the stream‐dwelling organisms but also to the local community of that landscape. Collaborative efforts involving the local communities and landowners of agroforests are necessary to conserve stream habitats in mixed‐use landscapes to sustain stream ecosystem services while providing conservation benefits to lesser‐known biodiversity.

## 5 Conclusion

This study adds to the existing knowledge of *Micrixalus kottigeharensis* by providing valuable insights into its habitat preference within the mixed‐use landscape of the Western Ghats. Our findings reveal that the species' count is shaped by an interplay of hydrological features, microclimatic conditions and land use, highlighting the role of both local stream characteristics and broader landscape context in determining its distribution. However, given the climate change crisis, alterations to stream habitats are likely to pose serious threats to stream organisms (Kakouei et al. 2018; Luedtke et al. 2023). These effects are compounded by intensifying anthropogenic pressures in mixed‐use landscapes that can lead to stream habitat degradation and ultimately lead to local extinctions. We recommend the retention of at least narrow strips of riparian vegetation along the streams, which can serve as a buffer and help maintain suitable microhabitat conditions for the amphibians. Conservation measures aimed at preserving critical habitats and restoring degraded corridors will be essential to sustain *M. kottigeharensis* populations within human‐use landscapes, given its extensive distribution outside the formal PA network.

## Author Contributions

**N. V. Rajiv:** conceptualization (equal), data curation (lead), formal analysis (lead), investigation (lead), methodology (lead), project administration (lead), resources (lead), software (lead), supervision (equal), visualization (lead), writing – original draft (lead), writing – review and editing (lead). **Vishnupriya Sankararaman:** conceptualization (equal), methodology (supporting), supervision (equal), writing – review and editing (supporting). **Vivek Ramachandran:** conceptualization (equal), methodology (supporting), resources (lead), supervision (lead), writing – review and editing (supporting).

## Funding

This work was supported by On The Edge Conservation, UK, On The Edge Fund.

## Conflicts of Interest

The authors declare no conflicts of interest.

## Data Availability

The data that support the findings of this study are openly available in figshare at https://figshare.com/s/4b96d52a2a031767aeff (Rajiv 2025).
